# In Vitro and in Vivo Efficacy of NBDHEX on Gefitinib-resistant Human Non-small Cell Lung Cancer

**DOI:** 10.7150/jca.46461

**Published:** 2020-10-18

**Authors:** Huanhuan Sha, Shuchen Dong, Chen Yu, Renrui Zou, Yue Zhu, Ya Lu, Junying Zhang, Haixia Cao, Dan Chen, Jianzhong Wu, Jifeng Feng

**Affiliations:** 1 The Affiliated Cancer Hospital of Nanjing Medical University, Jiangsu Cancer Hospital, Jiangsu Institute of Cancer Research, Baiziting42, Nanjing 210009, China; 2 The Forth Clinical School of Nanjing Medical University, Nanjing, 210009, China

**Keywords:** NBDHEX, gefitinib-resistant, NSCLC

## Abstract

Gefitinib, a first-generation EGFR tyrosine kinase inhibitor (EGFR-TKI), is recommended for treatment of non-small cell lung cancer (NSCLC) patients who harbor activating EGFR mutations. However, the tumors of most patients initially sensitive to gefitinib will develop resistance within several months of therapy. Drug resistance is a major obstacle to NSCLC treatment. The novel glutathione transferase P1 (GSTPi) inhibitor 6-(7-nitro-2, 1, 3-benzoxadiazol-4-ylthio) hexanol (NBDHEX) has recently been shown to be active against tumors. In this study, we investigated the *in vitro* and *in vivo* efficacy of NBDHEX against NSCLC. Treatment with NBDHEX inhibited GSTpi enzymatic activity and promoted apoptosis of gefinitb-resistant NSCLC cells. Moreover, NBDHEX reduced tumor growth in mice. These findings indicated that NBDHEX is a good candidate for treatment of NSCLC patients, and that NBDHEX offers a new approach to cancer therapy.

## Introduction

Lung cancer is the most common cancer in men and is the leading cause of cancer-related mortality worldwide 1. Among the several different pathologic types of lung cancer, non-small cell lung cancer (NSCLC) is the most common, accounting for approximately 80% of all lung cancer 2. About 10% of NSCLC patients carry a mutation in the tyrosine kinase domain of the epidermal growth factor receptor (EGFR) gene. Those patients have a rapid clinical response to EGFR tyrosine kinase inhibitors (TKIs) such as gefitinib 3-5. Nonetheless, most patients who are initially sensitive to gefitinib will develop resistance within 9-16 months of therapy 5, 6. Drug resistance is a major obstacle to NSCLC treatment. Multiple gefitinib resistance mechanisms include: mutation of T790M in exon 20 of EGFR, MET amplification, PIK3CA mutation, and others 7-10. Glutathione *S*-transferases (GSTs) are a multigene family of enzymes involved in xenobiotic detoxification, catalyzing the conjugation of GSH (glutathione) to carcinogens, drugs, toxins, as well as products of oxidative stress 11-14. GSTs, and in particular the isoenzyme GSTPi, have been shown to be overexpressed in many human cancer cell lines with the GST/GSH system contributing directly to drug resistance in some tumor cell types via detoxification 11, 15-17. More recently, GSTpi has been shown to associate with the c-Jun N-terminal kinase (JNK) complex and tumor necrosis factor receptor-associated factor 2 (TRAF2), which prevents the MAPK/JNK signaling cascade necessary to apoptosis 18, 19.

A variety of GST inhibitors have been reported that limit GSTpi. Among them, NBDHEX possesses a high degree of anti-proliferative activity, which depends upon its ability to arrest the cell cycle and trigger apoptosis in several human cancer cell lines. Mechanistically, NBDHEX inhibits GST's catalytic activity, although it does not function as a substrate for export pumps 20-22. Furthermore, in cancer cells, NBDHEX disrupts interaction of GSTP1-1 with key signaling effectors, which are essential for apoptosis and cell cycle effects 18, 23. Recently, NBDHEX was shown to act as a late phase autophagy inhibitor, which provides a new avenue by which to explore its therapeutic potential 24. We previously summarized NBDHEX's promising anticancer effects on various cancer cell lines and in animal models of osteosarcoma, small cell lung cancer, and acute myeloid leukemia 25. However, the effect of NBDHEX on GSTpi in gefitinib-resistant NSCLC remains unexplored.

Herein, we performed experiments initially aimed at evaluation of the effect of NBDHEX on gefitinib-resistant NSCLC. We found that treatment with NBDHEX decreased GSTpi's enzymatic activity and promoted apoptosis of gefitinib-resistance lung cancer *in vitro* and *in vivo*.

## Materials and Methods

### Cell lines

Human NSCLC cell line, HCC-827, was purchased from the Cell Bank of the Chinese Academy of Sciences (Shanghai, China). The gefitinib-resistant cell line, HCC827/GR, was maintained in our laboratory and used as a gefitinib-resistance model. Both cell lines were maintained in RPMI 1640 (Thermo Fisher Scientific, Massachusetts, MA, USA) supplemented with 10% fetal bovine serum (FBS) (Thermo Fisher Scientific), 80 U/mL penicillin, 0.08 mg/mL streptomycin (KeyGEN BioTECH, Jiangsu, China), and incubated in a humidified, 5% CO_2_ atmosphere, at 37°C.

### Drugs

Gefitinib was purchased from AstraZeneca (South San Francisco, CA, USA). NBDHEX (20 mM) stock solution were kindly provided by Dr. Luolan from State Key Laboratory of Pharmaceutical Biotechnology, School of Life Sciences, Nanjing University. It was prepared by dissolving the drug in dimethyl sulfoxide (DMSO) (Amresco, Solon, OH, USA). Just before use, NBDHEX was diluted to the appropriate concentration with the final DMSO concentration not exceeding 0.05% to 0.1%, dosages at which DMSO has no cytotoxic effect. For both drugs, working concentrations were prepared by diluting stock solutions in RPMI 1640 cell culture medium.

### Cell viability test

An evaluation of cell viability at different drug concentrations was made by Cell Counting Kit-8 (CCK-8) assay (Dojindo, Nagasaki, Japan). The cells were seeded in 96-well plates at a density of 5 × 10^4^/mL. After 24 h, various concentrations of NBDHEX were added. Seventy-two h later, 10 μL of CCK-8 was added to each well, and the plates were incubated for another 2 h. The absorbance of each well was measured at 450 nm using SpectraMax (Molecular Devices, San Jose, CA, USA). Drug dose-response curves were obtained and drug sensitivities of cell lines expressed as the half maximal inhibitory concentration (IC_50_).

### Enzyme Activity

GST activity was measured with a GST activity assay kit (KeyGEN BioTech, Nangjing, China) based on the manufacturer's instructions. Total cell lysates prepared as follows: cancer cell lines were collected by centrifugation at 800 g for 5 minutes at 4°C and washed twice in PBS. Cells were resuspended in PBS and lysated by a 10-second sonication. Lysates were centrifuged at 13,000 g for 10 minutes at 4°C and aliquots of the supernatant were used to measure the GST activity. GST activity was determined spectrophotometrically at 412 nm. One unit of GST activity was defined as the amount of enzyme that was able to reduced 1 μM of GSH per minute at 37°C.

### Western blotting analysis

Total protein from cells was harvested using radioimmunoprecipitation assay (RIPA) lysis buffer (Beyotime Biotechnology, Shanghai, China) by the manufacturer's instruction. Equal amounts of proteins were loaded onto 10%-12% sodium dodecyl sulfate (SDS) polyacrylamide gels and transferred onto polyvinylidene difluoride (PVDF) membranes (Merck Millipore, Darmstadt, Germany). The membranes were blocked with 5% skim milk for 2 h and then probed with primary antibodies. Anti-GSTpi, Bax, Bcl2, cleaved caspase 3, GAPDH, and Lamin B (1: 1,000) antibodies were incubated at 4°C overnight. IRdye 680 conjugated IgG secondary antibodies (1:5,000) were added and incubated for 1 h. An Odyssey infrared imaging system (LI-COR Biosciences, Lincoln, NE, USA) was used for visualization.

### Cytosolic and nuclear protein preparations

Cytosolic and nuclear proteins were extracted with a KeyGEN BioTECH Corp. Ltd extraction kit based on the manufacturer's instructions. Cells were collected and washed twice with cold phosphate-buffered solution (PBS) (KeyGEN BioTECH) at 800 × g for 3 min. All procedures were carried out at 4°C. Protein concentration was measured with the bicinchoninic acid protein assay reagent (Beyotime Biotechnology) with a Nanodrop 2000 spectrophotometer (ThermoFisher Scientific).

### Immunofluorescence confocal laser scanning microscopy

Prepared cells grown on glass bottom culture dishes (NEST Biotechnology, JIangsu, China) were fixed with 4% paraformaldehyde for 30 min, permeabilized with 0.5% Triton X-100 (Amresco) for 20 min and blocked with 5% bovine serum albumin (BSA) (Thermo Fisher Scientific) for 1 h. The specimens were incubated with primary GSTpi-antibody at 1:200 in blocking buffer overnight. Cells were then treated with secondary fluorescence-conjugated antibody at 1:1,000 in 5% BSA for 1 h and then washed with PBS (KeyGEN BioTECH). Nuclei were stained with 0.1 μg/mL DAPI (Thermo Fisher Scientific) for 5 min. Slides were imaged using a confocal laser microscope system (Leica, Wetzlar, Germany).

### Flow cytometry

HCC827/GR cells were seeded in each well of a 6-well plate and cultured for 24 h, after which 0, 4, 8, or 16 μM NBDHEX was added. After a 24 h incubation, the cells were washed with PBS and 400 μL of 1× binding buffer was added with 5 μL of Annexin V-FITC/PI (KeyGEN BioTECH) in the dark. Cells were analyzed with a flow cytometer (FACS Calibur, BD Biosciences, San Jose, CA, USA).

### Immunohistochemistry (IHC) staining

Tissues from NSCLC patients who were sensitive or resistant to gefitinib were collected. IHC staining was performed on formalin-fixed, paraffin-embedded sections. Sections were incubated with anti-GSTpi (1:100; Proteintech, Rosemont, IL, USA) antibody overnight at 4°C, followed by incubation with secondary antibody for 30 min. Sections were evaluated by a pathologist who was unaware of clinical treatment.

### Animal models

Male BALB/c nude mice (4-6-weeks-old) were purchased from the Shanghai Experimental Animal Center, China Academy of Science (Shanghai, China). Mice were maintained in the Animal Center of Nanjing Medical University and were housed in cages with automatically controlled temperature (22 ± 2°C), relative humidity (50-60%), and light (12 h light/dark cycles). All procedures were approved by the Animal Care and Use Committee of Nanjing Medical University, China. After acclimatization for 1 week, mice received a 200 μL HCC827/GR cells suspensions (5 × 10^7^ cells per mL) by subcutaneous injection in the back. After tumor size reached approximately 60-80 mm^3^, mice were randomly divided into four groups. NBDHEX was dissolved in dimethyl sulfoxide and diluted in saline. Each group was composed of five mice that were treated with 0.3, 0.5 or 1.0 mg/kg/d of NBDHEX administered *per os*. Control mice were always injected with drug vehicle. Animal body weight was monitored every 2 days. When experiment was complete, tumors were collected immediately and measured.

### Ethics statement

All tissues were collected from Nanjing Medical University Affiliated Cancer Hospital. All patients study provided written informed consent before use of tissue. The study was conducted in accordance with the standards of the Declaration of Helsinki and consistent with current ethical guidelines, which were approved by the Ethic Committee of the Nanjing Medical University.

### Statistical analysis

All independent experiments were carried out at least three times. Data are presented as means ± SD with statistical analysis performed with GraphPad Prism 6.0. Statistical analysis was by Student's *t*-test or the analysis of variance (ANOVA). The criterion for statistical significance was P < 0.05.

## Results

### GSTPi level and location in gefitinib-sensitive and resistant NSCLC tissues

**Figure 1:**
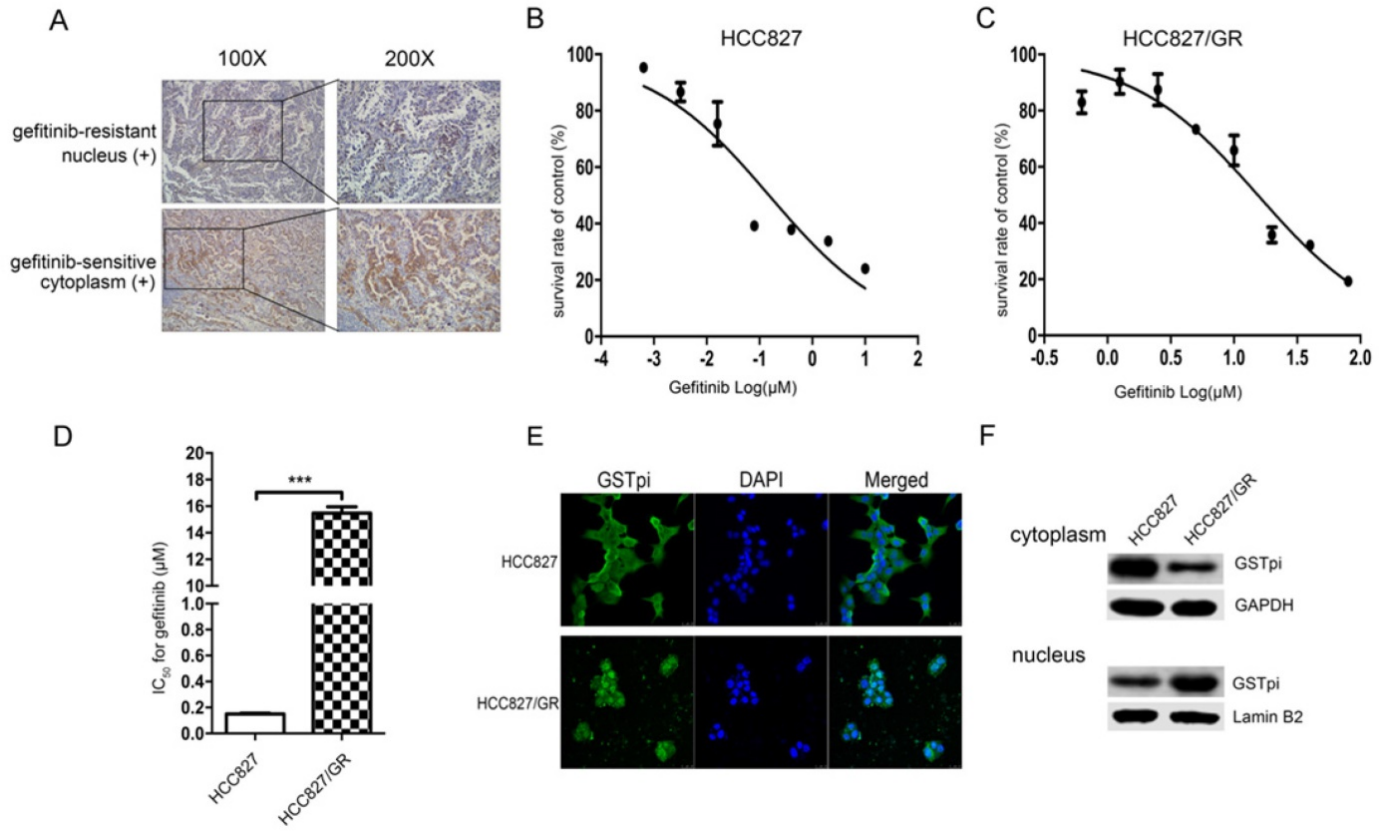
Gefitinib resistance related to the accumulation of GSTpi in the nucleus. (a) GSTpi expression and location in gefitinib-sensitive or resistant NSCLC tissues. (b) The gefitinib IC50 values of HCC827 and (c) HCC827/GR cell lines. (d) The fold increase in gefitinib resistance in the HCC827/GR cell line compared to the HCC827 cell line. (e) Immunofluorescent confocal laser scanning microscopy of GSTpi protein in HCC827/GR and HCC827 cell lines. (f) Western blot analysis of GSTpi protein in HCC827/GR and HCC827 cell lines. ^***^P < 0.001.

IHC staining showed that the level of GSTpi in gefitinib-resistant tissues was either similar or greater than that of gefitinib-sensitive tissues. GSTpi was enriched in the nucleus of gefitinib-resistant tissues and in the cytoplasm of gefitinib-sensitive tissues. These results suggested gefitinib resistance to be closely associated with GSTpi in the nucleus of NSCLC tissues (Fig. 1A).

### Characterization of gefitinib-resistant variants

We previously established EGFR-TKI-resistant cell lines. Gefitinib IC_50_ values and the fold increase in resistance for HCC827/GR are shown in (Fig. 1). The gefitinib IC_50_ values of the HCC827 cell line and the HCC827/GR cell line were 150 ± 8 × 10^-3^ μM and 15.47 ± 0.39 μM, respectively (Fig. 1B and 1C). Compared with the parental HCC827 cell line, the HCC827/GR cell line was 103.13-fold more resistant to gefitinib (Fig. 1D). The level and location of GSTpi protein in the two cell lines are shown in Fig. 1E. GSTpi was enriched in the nuclei of HCC827/GR but found in the cytoplasm of HCC827. Results were confirmed by western blotting (Fig. 1F). These findings demonstrated resistance to gefitinib to be associated with increased GSTPi protein in nuclei.

### Effect of NBDHEX on GSTpi enzyme activity and HCC827/GR cell viability

**Figure 2:**
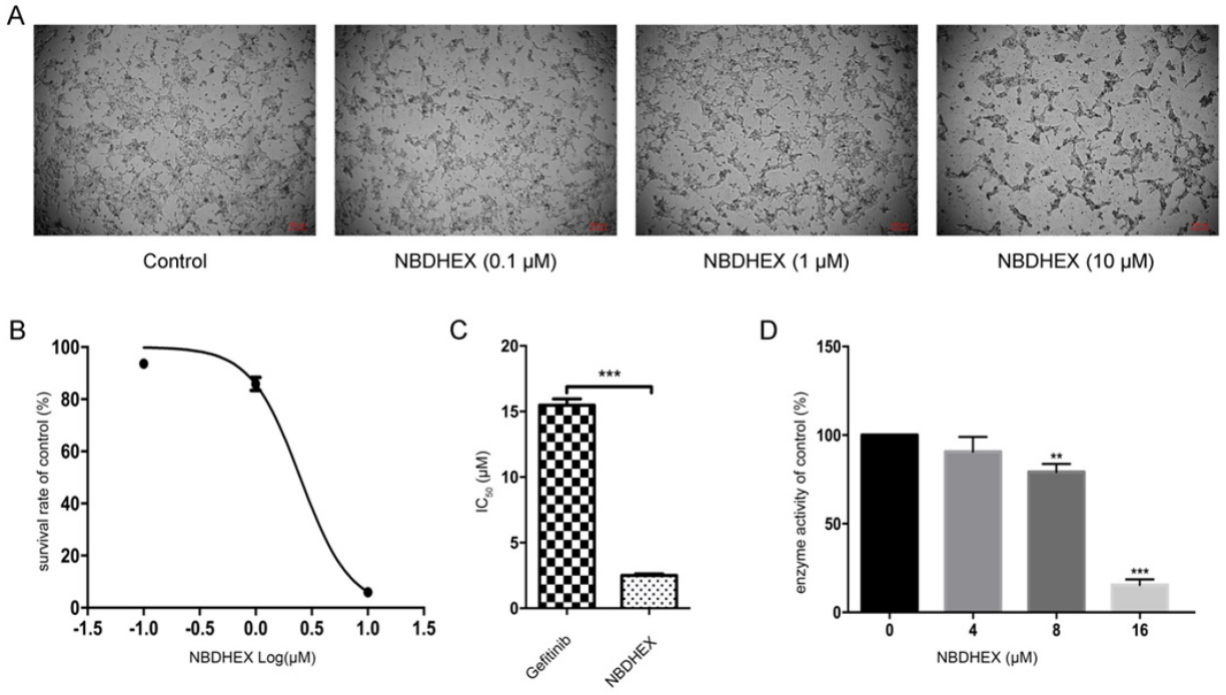
NBDHEX suppressed HCC827/GR growth and GSTpi enzymatic activity. (a) Changes in cell growth and morphology as judged by microscopy. (b) Survival curve of the HCC827/GR cell line after NBDHEX treatment. (c) NBDHEX and gefitinib IC50 values for the HCC827/GR cell line. (d) GSTpi enzymatic activity after treatment with NBDHEX. ^**^P < 0.01; ^***^P < 0.001.

**Figure 3:**
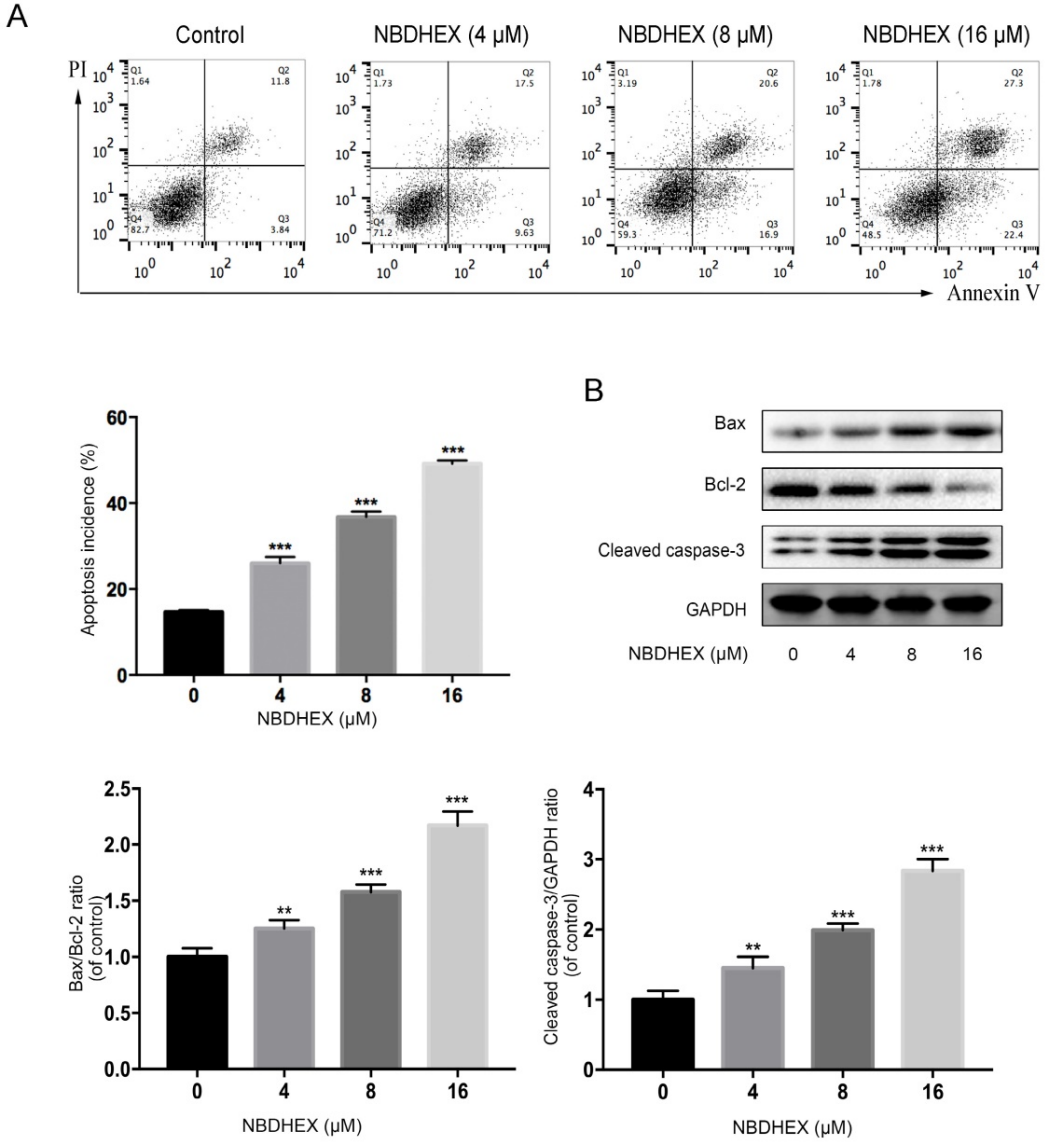
NBDHEX inhibited HCC827/GR cell viability by promoting apoptosis of HCC827/GR. (a) The percentage of apoptotic cells determined by flow cytometric analysis. (b) Western blot analysis of cellular bax, bcl-2, and cleaved caspase-3 after treatment with NBDHEX. GAPDH was used as an internal control. ^**^P < 0.01; ^***^P < 0.001.

To assess the effect of NBDHEX on HCC827/GR cells and the enzymatic activity of GSTpi, HCC827/GR cells were treated with increasing concentrations of NBDHEX. Results showed NBDHEX to significantly suppress HCC827/GR cell line growth with an IC_50_ value of 2.5 ± 0.09 μM (Fig. 2A and 2B). HCC827/GR was more sensitive to NBDHEX than to gefitinib (Fig. 2C). The effect of NBDHEX on GST activity was determined with a GSTpi enzyme activity kit. NBDHEX was found to inhibit greater than 75% of GSTpi's enzymatic activity when the concentration was greater than 16 μM (Fig. 3D). Therefore, NBDHEX suppressed HCC827/GR growth in part through inhibition of GSTpi enzymatic activity.

### Effect of NBDHEX on HCC827/GR apoptosis

As judged by flow cytometry, NBDHEX promoted apoptosis of the HCC827/GR cell line (Fig. 3A). Moreover, western blot analysis showed that apoptosis-associated proteins, bax and caspase-3, were increased significantly with NBDHEX treatment (Fig. 3B). In contrast, the anti-apoptotic protein, bcl-2, was markedly decreased with NBDHEX treatment. These results demonstrated that NBDHEX reduced HCC827/GR cell viability, in part, by promotion of HCC827/GR apoptosis.

### In vivo studies

**Figure 4:**
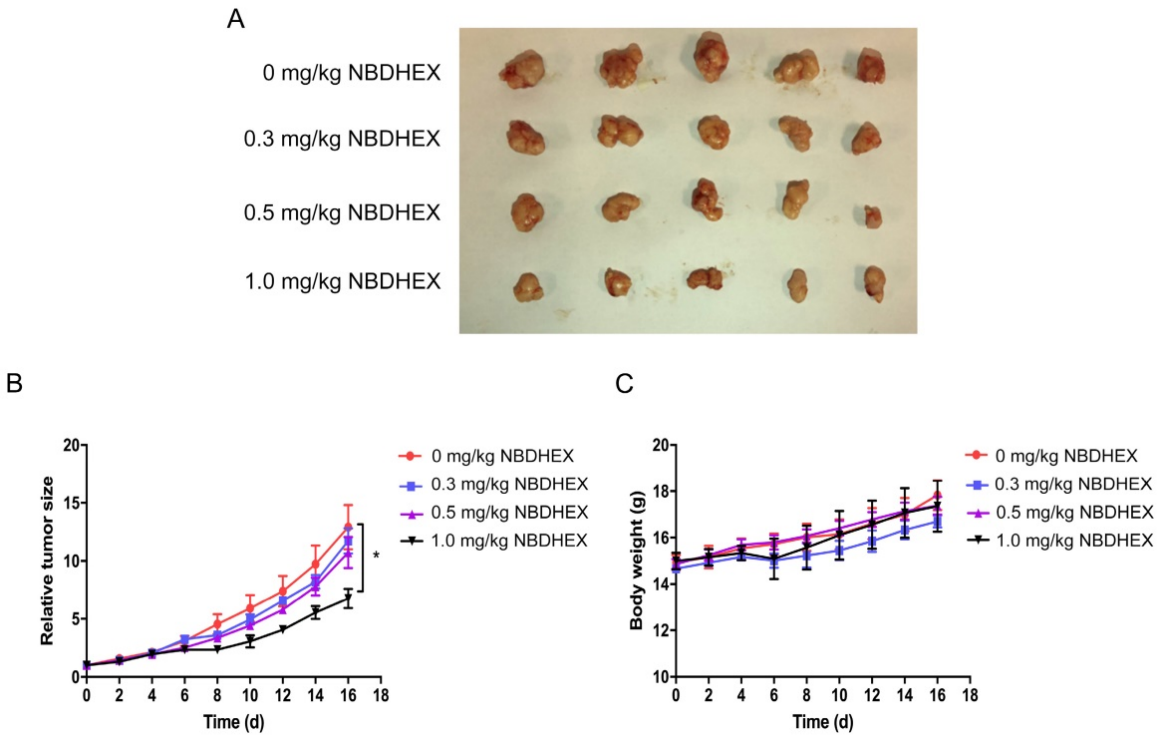
NBDHEX inhibited tumorigenesis *in vivo*. (a) Total number of mouse tumors. (b) Tumor volumes and (c) mouse weight calculated 2 days after inoculation. The data are means ± S.D. ^*^P < 0.05.

The *in vivo* anti-tumor efficacy of NBDHEX was evaluated in mice. After daily treatment for 16 d, statistically significant tumor inhibition (approximately 50%) was observed when the NBDHEX concentration reached 1.0 mg/kg/d (Fig. 4A and 4B). At sacrifice, NBDHEX was well tolerated with no reduction in mouse body weight (Fig. 4C).

## Discussion

Worldwide lung cancer is the most common malignant tumor with the highest mortality rate 1. Although surgery, radiotherapy and chemotherapy have improved outcomes, the overall 5-year survival rate for lung cancer is only 15%. NSCLC accounts for 80-85% of all lung cancer 2, 26. Overactivation of EGFR is an important driver in the development of lung cancer due to activation of a series of downstream events that trigger multiple signaling pathways.

These pathways result in proliferation of cancer cells, inhibition of apoptosis, and promotion of metastasis 27-29. Recently, therapy that targets EGFR has become an efficacious clinical treatment for NSCLC. The National Comprehensive Cancer Network (NCCN) guidelines have recommended EGFR tyrosine kinase inhibitors (EGFR-TKI) as first-line therapeutic agents for advanced NSCLC patients with EGFR sensitive mutations 3, 4. However, these NSCLC patients acquire drug resistance 9 to 16 months after treatment 4-6, 30. The main causes of drug resistance are the T790M mutation in exon 20 of EGFR, MET amplification, and PIK3CA mutation 7, 9, 10, 31-35. Continuous EGFR activation promotes the proliferation and migration of NSCLC cells. Recently, a number of new drugs have been developed to overcome EGFR-TKI resistance, with a few drugs achieving positive clinical results in some populations 36-39. EGFR-TKI drug resistance mechanisms are multiple and not fully understood. Therefore, exploring new targets and new mechanisms of EGFR-TKI resistance are key to effective treatment of NSCLC.

GSTpi over expression has been well documented to induce cancer cell resistance to a variety of anticancer drugs. We have explored the relationship between GSTpi expression and gefitinib resistance in NSCLC patients' tissues. We found that GSTpi expression was significantly localized to the nuclei of cells within recurrence foci (biopsy specimen of patients with drug resistance), but mainly distributed in the cytoplasm in cells within the primary foci (initial surgical specimen) of the same patient. These findings suggested gefitinib resistance to be closely associated with nuclear GSTpi in NSCLC. GSTpi levels were evaluated in two cell lines HCC827 and HCC827/GR by western blotting and immunofluorescence. Results demonstrated GSTpi to be enriched in the nuclei of the resistant HCC827/GR cell line and in the cytoplasm of the gefitinib-sensitive HCC827 cell line. These results demonstrated the location of GSTpi to be closely associated with gefitinib resistance in NSCLC.

Based on evaluations of GSTpi in many cancer lines, a few GSTpi inhibitors have been designed 21, 40, 41. Among these, NBDHEX has been shown recently to be a promising anticancer drug 20, 24, 42, 43. However, the usefulness of NBDHEX in NSCLC gefitinb-resistance has been unexplored 25. To evaluate the effects of NBDHEX, HCC827/GR cells were treated with various concentrations of the drug. Results showed NBDHEX to significantly suppress growth of the HCC827/GR cell line with an IC_50_ value of 2.5 ± 0.09 μM. This IC_50_ value is much lower than the IC_50_ for gefitinib. Hence, NBDHEX may be more effective than gefitinib for treatment of NSCLC.

The most widely evaluated function of GSTs is the interaction of reduced GSH with several electrophilic compounds, both endogenous and xenobiotic. Many anticancer drugs are substrates for GSTs and their interaction with GSH can be catalyzed efficiently with the product extruded from the cell by specific export pumps 44-46. The detoxifying activity of GSTs plays a significant role in drug resistance of some tumor cell types via the activation of GST/GSH 16, 44. Interestingly, in some cancer cell lines, enhanced GSTP1 enzymatic activity is very often associated with an increase in GSTP1 gene expression, increased GSTP1-1 protein level, or both. Additionally, an increase in both intracellular levels and enzymatic activity of GSTP1-1 is closely related to the degree of cisplatin resistance 22. Based on these observations, we investigated whether the effectiveness of GSTpi was due to its enzymatic activity. NBDHEX was found to inhibit GSTpi enzymatic activity at concentrations more than 16 μM. However, lower doses of NBDHEX did significantly affect HCC827/GR cells, suggesting other mechanisms of action. NBDHEX is demonstrated to bind the H-site of GSTpi and form a complex with GSH. It leads to the activation of the pro-apoptotic pathway via disrupting the interaction between the GSTpi and key signaling effectors, namely TRAF2 and JNK 18, 19, 47. Furthermore, NBDHEX promoted a caspase-dependent apoptosis which is unusual for P-glycoprotein (P-gp) overexpressing cells, wherein the apoptotic pathway is a direct consequence of the dissociation of GSTP1-1 from JNK 42. Hence, apoptosis was demonstrated by flow cytometry and detection of apoptosis-related proteins in this study. The results showed that NBDHEX promoted apoptosis. Therefore, NBDHEX could suppress HCC827/GR growth, in part, through promotion of apoptosis. Because there are few reports of GSTpi's function in the nucleus, we will continue to explore the relationship between GSTpi in the nucleus and gefitinib resistance in NSCLC.

In conclusion, we demonstrated NBDHEX to be capable of inhibiting growth and promoting apoptosis of gefitinib-resistance NSCLC both *in vitro* and *in vivo*. These observations suggested NBDHEX to be an appropriate candidate for the treatment of NSCLC.

## Abbreviations

EGFR: epidermal growth factor receptor
TKIs: tyrosine kinase inhibitors
NSCLC: non-small cell lung cancer
GSTpi: glutathione transferase P1
NBDHEX: 6-(7-nitro-2, 1, 3-benzoxadiazol-4-ylthio) hexznol
JNK: c-Jun N-terminal kinase
TRAF2: tumor necrosis factor receptor-associated factor 2
FBS: fetal bovine serum
DMSO: dimethyl sulfoxide
CCK-8: cell counting kit-8
IC50: half maximal inhibitory concentration
SDS: sodium dodecyl sulfate
PVDF: polyvinylidene difluoride
BSA: bovine serum albumin
PBS: phosphate-buffered saline
NCCN: national comprehensive cancer network
P-gp: P-glycoprotein
